# LncRNA AFAP1-AS1 promotes tumorigenesis and epithelial-mesenchymal transition of osteosarcoma through RhoC/ROCK1/p38MAPK/Twist1 signaling pathway

**DOI:** 10.1186/s13046-019-1363-0

**Published:** 2019-08-23

**Authors:** Deyao Shi, Fashuai Wu, Shidai Mu, Binwu Hu, Binlong Zhong, Feng Gao, Xiangcheng Qing, Jianxiang Liu, Zhicai Zhang, Zengwu Shao

**Affiliations:** 1 Department of Orthopaedics, Union Hospital, Tongji Medical College, Huazhong University of Science and Technology, 1277 Jiefang Road, Wuhan, 430022 China; 2 Institute of Hematology, Union Hospital, Tongji Medical College, Huazhong University of Science and Technology, 1277 Jiefang Road, Wuhan, 430022 China

**Keywords:** Long non-coding RNA, AFAP1-AS1, Osteosarcoma, Epithelial-mesenchymal transition, Twist1, RhoC

## Abstract

**Background:**

An increasing number of studies have demonstrated that long non-coding RNAs (lncRNAs) play pivotal roles in cancer onset and development. LncRNA AFAP1-AS1 has been validated to be abnormally upregulated and play oncogenic roles in various malignant tumors. The biological role and mechanism of AFAP1-AS1 in OS (osteosarcoma) remains unclear.

**Methods:**

Quantitative reverse transcription PCR (qRT-PCR) is applied to examine AFAP1-AS1 expression in OS tissues and OS cell lines. The function of AFAP1-AS1 in OS cells is investigated via in-vitro and in-vivo assays. Western bolt and rescue experiments are applied to detect the expression changes of key molecules including epithelial-mesenchymal transition markers and identify the underlying molecular mechanism. RNA immunoprecipitation is performed to reveal the interaction between AFAP1-AS1 and RhoC.

**Results:**

AFAP1-AS1 expression is upregulated in human OS tissues and cell lines. AFAP1-AS1 knockdown induces OS cell apoptosis and cell cycle G0/G1 arrest, suppresses OS cells growth, migration, invasion, vasculogenic mimicry formation and epithelial-mesenchymal transition (EMT), and affects actin filament integrity. AFAP1-AS1 knockdown suppresses OS tumor formation and growth in nude mice. AFAP1-AS1 knockdown elicits a signaling inhibition including decreased levels of RhoC, ROCK1, p38MAPK and Twist1. Moreover, AFAP1-AS1 interacts with RhoC. Overexpression of RhoC can partly reverse AFAP1-AS1 downregulation-induced cell EMT inhibition.

**Conclusions:**

AFAP1-AS1 is overexpressed in osteosarcoma and plays an oncogenic role in osteosarcoma through RhoC/ROCK1/p38MAPK/Twist1 signaling pathway, in which RhoC acts as the interaction target of AFAP1-AS1. Our findings indicated a novel molecular mechanism underlying the tumorigenesis and progression of osteosarcoma. AFAP1-AS1 could serve as a promising therapeutic target in OS treatment.

**Electronic supplementary material:**

The online version of this article (10.1186/s13046-019-1363-0) contains supplementary material, which is available to authorized users.

## Background

Osteosarcoma (OS) is one of the most common malignant bone tumors, which occurs mostly in children and adolescents, with 10 to 25 years as the major onset age [1]. At present, the main treatment regimen for osteosarcoma is surgical resection combined with chemotherapy. According to recent research conclusions, osteosarcoma patients’ overall survival has been dramatically improved through widely used neoadjuvant and adjuvant chemotherapeutic regimens. For example, referring to the experience of COSS (the interdisciplinary Cooperative German-Austrian-Swiss Osteosarcoma Study Group), surgery and varying combinations of high-dose methotrexate with leucovorin rescue, doxorubicin, cisplatin, and/or ifosfamide and others multidrug chemotherapy were used in most protocols of osteosarcoma treatment [2, 3]. For osteosarcoma patients without distant metastasis, the five-year survival rate can reach 55–70% after standardized treatment, in which around 90% of patients can attain limb salvage. However, distant metastasis occurred in nearly 85% of the osteosarcoma patients at the initial treatment. For patients with early metastasis or chemo-resistance, even if treated with standard adjuvant chemotherapy and tumor resection, the five-year survival rate is approximately 5–20% [4–6]. Moreover, no breakthrough has been made in the fields of clinical and scientific research of osteosarcoma. Therefore, a better understanding of tumor biological behavior of osteosarcoma and deeper investigation of pivotal mechanism promoting osteosarcoma tumorigenesis and development are extremely important to intensify the treatment efficacy of osteosarcoma and further improve patients’ prognosis.

Epithelial to mesenchymal transition (EMT), a process that defined as cells changing their epithelial phenotype, losing cell polarity and transforming in to cells with mesenchymal characteristics, such as enhanced migratory and wandering ability [7, 8]. Recently, abundant evidences revealed that EMT occurs in osteosarcoma and associate with initiation, progression, and metastasis especially in osteosarcoma [9–11]. The physiologic processes of EMT occurs during various biological behaviors including embryogenesis, inflammation and repair of tissue injury. Loss of epithelial phenotype and the acquisition of mesenchymal properties are essential characteristics of EMT [8, 11]. With the development of research, critical roles of EMT in cancer development were discovered gradually, especially in the aspect of cancer metastasis. Molecular mechanisms that regulate EMT are complicated and not fully understood. Many factors may associate to EMT process, including the expression of EMT related transcriptional factors, such as Snail, Slug, Twist, ZEB, and activation level of some certain signaling pathways, such as TGF-β/Samd, Wnt/β-catenin, Hedgehog signaling pathway [11–13].

Long non-coding RNA (lncRNA) is a class of non-coding RNA of which the transcripts include more than 200 nucleotides. As the major member of non-coding RNAs, lncRNA has been revealed in many studies recently to act as potential regulators in various aspects of cell biological behavior including cell proliferation, programmed cell death, migration, differentiation [14, 15]. Different kinds of lncRNA were demonstrated to affect cell biological functions through diversified ways involving chromatin modification, gene transcriptional regulation, epigenetic regulation, mRNA post-transcriptional processing, interaction with proteins or microRNAs [16–18]. Furthermore, aberrant expression of lncRNA has been indicated to relate with a diverse range of diseases including malignancies [19]. Actin filament-associated protein 1-antisense RNA 1 (AFAP1-AS1) has been validated to be abnormally upregulated in various malignant tumors, such as nasopharynx carcinoma, colorectal cancer, and cholangiocarcinoma [20]. Moreover, there is statistically significant association between high expression of lncRNA AFAP1-AS1 and malignant clinicopathological features according to recent oncology studies involving colorectal cancer and lung adenocarcinoma [21–23]. According to Li et al.’s study, AFAP1-AS1 was overexpressed in osteosarcoma and high expression of AFAP1-AS1 was associated with poor prognosis of osteosarcoma patients [24]. However, the functional roles and underlying regulatory mechanism of AFAP1-AS1 have not been investigated thoroughly in osteosarcoma. In our study, we detected the expression level of lncRNA AFAP1-AS1 in the OS tissues and the adjacent non-tumor tissues via, as well as in the osteosarcoma cell lines and normal osteoblasts cell line. Relevant molecular mechanism was further investigated.

## Materials and methods

### Patients and osteosarcoma samples

This study was approved by the Research Ethics Committee of Huazhong University of Science and Technology. Informed consents were obtained from all patients or their guardians. Osteosarcoma tissue samples and their matched adjacent non-tumor tissues (eight pairs) were collected from patients who underwent operation at Huazhong University of Science and Technology Affiliated Wuhan Union hospital in 2018. In this study, no patient received chemotherapy before surgical operation (Detailed clinical information was recorded in Additional file 3: Table S2). Tissue samples were collected during definite surgery and stored in liquid nitrogen before usage. All patients were diagnosed with osteosarcoma according to histopathological examination.

### Cell lines and cell culture

Four human osteosarcoma cell lines (MNNG/HOS, MG63, SaOS-2 and U2OS) and normal human osteoblasts cell line (hFOB 1.19) were obtained from the Cell Bank of China Academy of Sciences (Shanghai, China). MNNG/HOS, MG63 and U2OS cells were cultured in α-MEM medium (HyClone, USA) supplemented with 10% FBS (Gibco, USA). SaOS-2 cells were cultured in RPMI-1640 medium (Gibco, USA) supplemented with 15% FBS. hFOB1.19 cells were cultured in Ham’s F12/Dulbecco’s modified Eagle’s medium (DMEM, Gibco) supplemented with 10% FBS, 100 U/mL penicillin and 100 mg/mL streptomycin (Invitrogen, Grand Island, NY, USA). Four human osteosarcoma cell lines were maintained in a humidified incubator at 37 °C with a 5% CO2 atmosphere. hFOB 1.19 cell line were maintained at 33.5 °C with a 5% CO2 atmosphere.

### RNA isolation and qRT-PCR

Total RNA was extracted from tissues or cultured cells using TRIzol reagent (Invitrogen, Grand Island, NY, USA). Total RNA (1 μg) was reverse transcribed to cDNA in a final volume of 20 μL using random primers under standard conditions with the PrimeScript RT Reagent Kit (Takara, Dalian, China). Real-time PCR analyses were carried out using SYBR Premix Ex Taq (Takara) according to the manufacturer’s instructions. Results were normalized to the expression of glyceraldehyde 3-phosphate dehydrogenase (GAPDH), and data were collected based on the comparative cycle threshold (CT) (2^-ΔΔCT^) method. The primer sequences for PCR were shown in Additional file 2: Table S1.

### Small interfering RNA transfection

For the RNA interference, four different small interfering RNAs (siRNAs) that targeted AFAP1-AS1 RNA were designed to lower off-target effects. All siRNAs and control siRNA were purchased from Genepharma Company (Shanghai, China). The sequences of siRNAs that targeted AFAP1-AS1, RhoC, Twist1 and scrambled (negative control) siRNA were shown in Additional file 2: Table S1. After test and verification, two siRNAs (#1 and #2) were considered to be effective for AFAP1-AS1 knockdown. Osteosarcoma cell lines were seeded in 6-well plates for 24 h then transfected with specific siRNAs using LipofectamineRNAi MAX in serum-free medium according to manufacturer’s protocols (Invitrogen, Grand Island, NY, USA). Cells were harvested 48 h after transfection for following assays.

### Plasmid DNA transfection

The full-length human RhoC and Twist1 cDNA expression plasmids (pENTER-RhoC and pENTER-Twist1) and the control plasmid pENTER vector were purchased from Vigene Biosciences (Jinan, China). MNNG/HOS and U2OS cells were transfected using Lipofectamine 3000 reagent (Invitrogen, Grand Island, NY, USA) according to the manufacturer’s instructions. Cells were harvested for following assays 48 h after transfection.

### Cell proliferation assay

Cell viability was assessed using a CCK-8 kit (Dojindo Laboratories Co. Ltd., Kumamoto, Japan). In brief, cells (3.0 × 10^3^ cells/well) after transfection were seeded onto 96-wellplates with 100 μL per well of α-MEM medium with 10% FBS. Each group set six replicates. At the indicated time points, the medium was replaced by 90 μL fresh α-MEM medium with 10 μL CCK-8 solution. After 1–4 h’ incubation at 37 °C, the absorbance was recorded at 450 nm. Cell viability was calculated according to the manufacturer’s instruction.

### Colony formation assay

Transfected cells (1.0 × 10^3^ cells/well) were trypsinized into a single-cell suspension and seeded into 6-wellplates and cultured for 2 weeks in medium supplemented with 10% FBS and every 3 days the medium was replaced. At the 14th day, cells were fixed with 4% paraformaldehyde for 15 min and then stained with 1% crystal violet. Clones containing more than 50 cells were counted using a grid. Three independent replicates were performed.

### Flow cytometric analysis

MNNG/HOS and U2OS cells were transfected and 48 h after transfection cells were harvested for analysis. For the apoptosis assessment, Annexin V-fluorescein isothiocyanate (FITC) assay was used to detect apoptosis by flow cytometry. After the double staining with FITC-Annexin V and propidium iodide (PI), the relative ratio of viable, dead, early apoptotic, and apoptotic cells was analyzed with a flow cytometry (FACScan, Becton Dickinson, Franklin, NJ). For the cell cycle assessment, transfected cells were washed with cold PBS for 2 times and fixed in 75% ethanol at − 20 °C overnight. After washed with PBS again, the cells were treated with RNaseA at 37 °C for 30 min and then stained with propidium iodide (BD, USA) at room temperature for 30 min in dark. The FACScan flow cytometer was applied to analyze the percentage of cells in G0/G1, S, and G2/M phase. The raw data was gated through FlowJo software 7.0 (Tree Star Inc., Ashland, USA).

### Western blot analysis

Cell protein content in the lysates was determined through the BCA protein assay (Beyotime Biotechnology, China) according to manufacturer’s instructions. Cells protein lysates were separated in 10% SDS-polyacrylamide gel electrophoresis (SDS-PAGE) and transferred to 0.22 μm PVDF membranes (Millipore, Massachusetts, USA). 5% skim milk containing 0.1% Tween-20 was used to block the PVDF membranes and then incubated with specific antibodies at 4 °C overnight. Horseradish peroxidase-linked secondary antibodies were added at a dilution ratio of 1:1000, and incubated at room temperature for 1 h. The immunoreactive bands were visualized using ECL Kit (Thermo Fisher). Anti-cleaved Caspase3, anti-Bcl-2, anti-Bax and anti-N-Cadherin antibodies were purchased from Abcam, USA. Anti-E-Cadherin, anti-Vimentin, anti-Cyclin D1, anti-MMP-9, anti-RhoC, anti-ROCK1, anti-p38MAPK, anti-Phospho-p38MAPK, and anti-GAPDH were purchased from Cell Signaling Technology, Inc. Anti-Twist1 and anti-AFAP1 antibodies were purchased from Proteintech Group, USA.

### Cell migration and invasion assays

The capacity of cell migration was measured on Transwell chamber plates, (24-well format, 8 μm pore size, BD Biosciences, St Louis, MO). Transwell chamber with pre-coated 100 μL Matrigel (1:7 dilutions, BD Biosciences, St Louis, MO) was applied for assessment of the capacity of cell invasion. Cells (5 × 10^4^) in 100μLserum-free medium were seeded onto the top chamber of Transwell. The bottom well containing 20% FBS was used as growth medium. After incubation at 37 °C for 36 h, cells that invaded through the pores of the Transwell were fixed with 4% paraformaldehyde for 15 min and then stained with 1% crystal violet. Transwell chamber after staining was observed under a microscope. The average number of migratory and invasive cells were counted from 5 random fields.

### In vivo assay

This study was carried out in strict accordance with the recommendations in the Guide for the Care and Use of Laboratory Animals of the National Institutes of Health. The protocol was approved by the Committee on the Ethics of Animal Experiments of Huazhong University of Science and Technology. Nude mice (BALB/c, female 5 to 6-week-old) were injected subcutaneously with 5 × 10^6^ MNNG/HOS cells. Tumor volumes were calculated from the length (a) and the width (b) via the following formula: volume (mm^3^) = ab^2^/2. All Mice were randomly divided to two groups and prepared for treatment with siRNA or as controls when the subcutaneous tumor volume reached 50 to 60 mm^3^. Cholesterol conjugated siRNA was used for in vivo RNA delivery. The siRNA and respective negative control were purchased from Ribobio (Guangzhou, China). For delivery of cholesterol conjugated siRNA, 10 nmol siRNA in 150 μL saline buffer was locally injected into the tumor mass every 3 days. Three weeks after injection, the animals were sacrificed, and tumors were harvested (measured and weighed) and fixed in 4% paraformaldehyde.

### Subcellular fractionation

For MNNG/HOS and U2OS cells, the separation of nuclear and cytoplasmic fractions was performed using the PARIS Kit (Ambion, Austin, Texas, USA) following the manufacturer’s instructions.

### RNA immunoprecipitation (RIP) assay

The EZ-Magna RIP kit (Millipore, Billerica, MA) was used to perform RIP assay according to the manufacturer’s protocol. In brief, about 2.0–2.5 × 10^7^ U2OS and MNNG/HOS cells at around 80% confluency were scraped off the culture plate, and treated with complete RIP lysis buffer. After Preparation of magnetic beads for immunoprecipitation, a total of 100 μL of the cell extraction supernatant was incubated with beads-antibody complex in RIP immunoprecipitation buffer overnight with rotating at 4 °C using antibodies against RhoC and control IgG (Millipore, Billerica, MA). Then all beads were washed with wash buffer, and the immunoprecipitate complex were incubated with Proteinase K Buffer at 55 °C for 30 min with shaking to purify RNA. Finally, immunoprecipitated RNA was analyzed by qRT-PCR.

### Immunohistochemistry assay

Immunohistochemistry for Ki67 and Vimentin was performed on the 4% paraformaldehyde-fixed, paraffin-embedded subcutaneous tumor tissues using a primary antibody against target antigen (anti-Ki67, Abcam, USA; anti-Vimentin, Cell Signaling Technology, Inc) and a horseradish peroxidase-conjugated IgG (Abcam, USA). The proteins in situ were visualized with 3,3-diaminobenzidine.

### Immunofluorescence assay

Cells were fixed in 4% paraformaldehyde for 20 min, permeabilized with 0.5% Triton X-100 for 5 min and blocked in phosphate-buffered saline (PBS) containing 5% fetal bovine serum for 30 min. Then, the cells were incubated for 1 h with phalloidin-FITC (Molecular Probes, Beyotime, China) followed by three washes with PBS and then stained with 4′, 6-diamidino-2-phenylindole (DAPI) for 10 min at room temperature.

### Tube formation assay

To assess the formation of vasculogenic mimicry (VM), the Matrigel tube formation assay was performed. In brief, 50 μL Matrigel (BD Biosciences, San Jose, CA, USA) was added to 96-well plate at 4 °C and allowed to solidify at 37 °C for 0.5 h. Then, cells suspended in 100 μL medium were seeded into the Matrigel-coated 96-well plate and incubated at 37 °C. The formation of capillary-like tubes was photographed at 1, 2, 4, 6, and 12 h after seeding. Quantitative analysis of the mean number of tube-like structures from five randomly chosen fields was carried out using the ImageJ software (National Institutes of Health, Bethesda, MD, USA).

### Statistical analysis

The SPSS 18.0 software (IBM, Chicago, IL) was used to perform statistical analysis in each experiment. Results were represented as means ± standard deviation. Student’s t-test (two-tailed) was used to examine significant difference between any two groups and one-way ANOVA was used to evaluate three or more groups. *P* < 0.05 was considered to be statistically significant.

## Results

### Expression of AFAP1-AS1 is upregulated in human OS tissues and cell lines

**Fig. 1:**
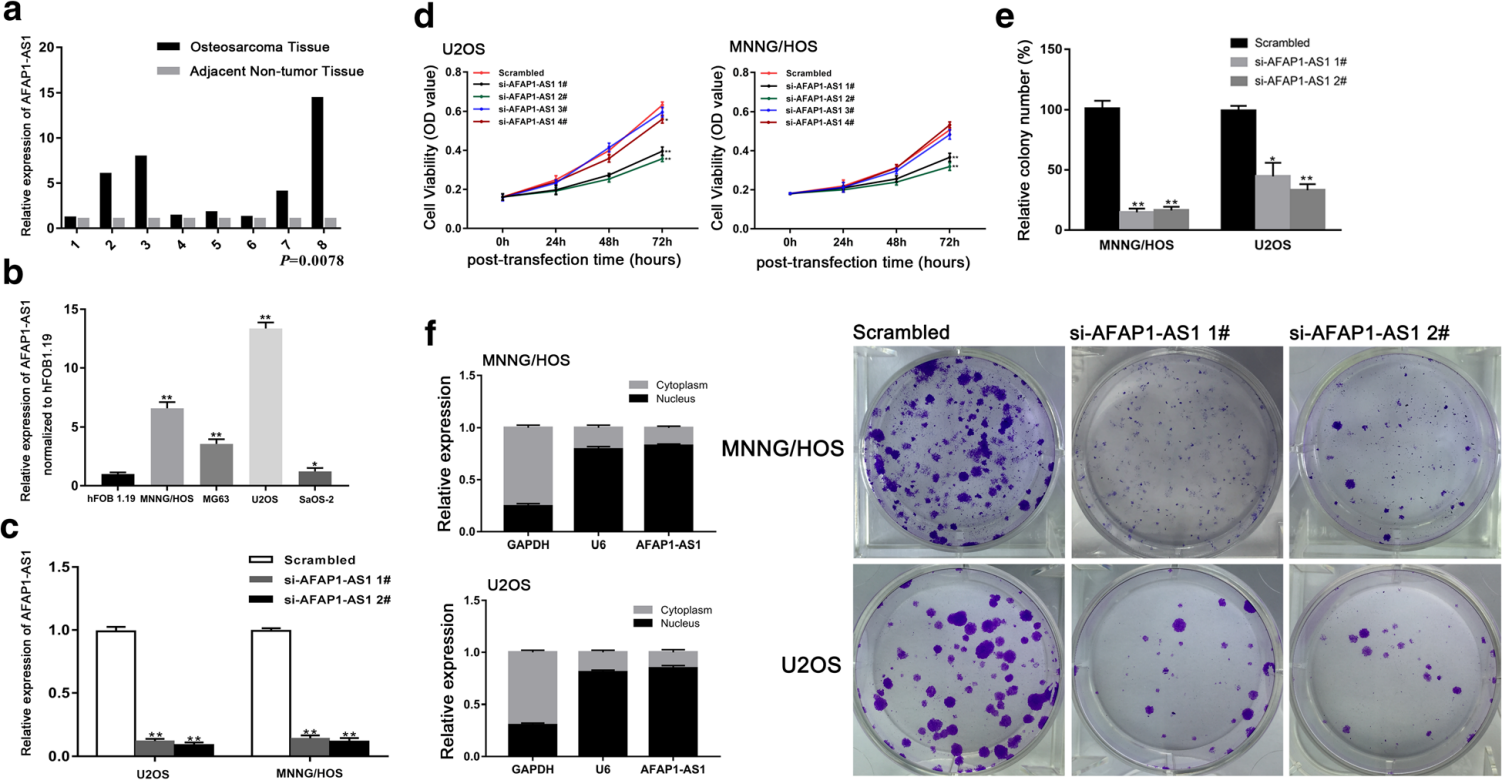
Relative AFAP1-AS1 expression in OS tissues and cell lines. The effects of AFAP1-AS1 on OS cell proliferation in vitro. **a** Expression of AFAP1-AS1 in OS tissue (*n* = 8) was significantly higher compared with adjacent non-cancerous tissues (*n* = 8), *P =* 0.0078. **b** Expression of AFAP1-AS1 in OS cell lines (MNNG/HOS, MG63, SaOS-2 and U2OS) was significantly higher compared with normal human osteoblasts cell line (hFOB 1.19). **c** Transfection with siRNA (si-AFAP1-AS1 1# and 2#) resulted in significantly lower AFAP1-AS1 expression. **d** After transfection, in the groups of si-AFAP1-AS1 1# and 2#, the OS cells (MNNG/HOS and U2OS) exhibited significantly decreased growth activity compared with the control. **e** Knockdown of AFAP1-AS1 significantly inhibited colony formation of MNNG/HOS and U2OS cells. **f** AFAP1-AS1 expression in nucleus was higher than in cytosol in OS cells (MNNG/HOS and U2OS). *P < 0.05, **P < 0.01

We firstly evaluated the transcript levels of AFAP1-AS1 in 8 cases of human OS tissues and their matched adjacent non-tumor tissues by qRT-PCR. AFAP1-AS1 was significantly overexpressed in OS tissues (Fig. 1a). We further assessed the expression level of AFAP1-AS1 in OS cell lines (MNNG/HOS, MG63, U2OS and SaOS-2) compared with human normal osteoblast cell line (hFOB1.19). AFAP1-AS1 was significantly over expressed in the four OS cell lines compared to hFOB1.19. MNNG/HOS and U2OS cell lines showed relative higher levels of AFAP1-AS1 expression (Fig. 1b). These results indicated that AFAP1-AS1 might play a pivotal role in OS tumorigenesis and development.

### Knockdown of AFAP1-AS1 inhibited OS cells proliferation

To explore the possible regulation effect of AFAP1-AS1 in OS cells, a total of four small interfering RNA (siRNA) targeted to AFAP1-AS1 and one scrambled siRNA (negative control siRNA) were applied. U2OS and MNNG/HOS cells were chosen for transfection. Forty-eight hours post-transfection we detected that two siRNAs exerted valid silencing effect. The expression of AFAP1-AS1 were knockdown by over 85% in MNNG/HOS cells and over 90% in U2OS cells compared with control group (Fig. 1c). CCK-8 assay demonstrated that MNNG/HOS and U2OS cell viability were significantly decreased after knockdown of AFAP1-AS (Fig. 1d). In addition, knockdown of AFAP1-AS1 significantly inhibited colony formation MNNG/HOS and U2OS cells (Fig. 1e). These results indicated AFAP1-AS1 played an oncogenic role in OS cells proliferation. Besides, in both MNNG/HOS and U2OS cell lines, we identified that AFAP1-AS1 expression in nucleus was higher than in cytosol, as shown in Fig. 1f.

### Knockdown of AFAP1-AS1 induced OS cells apoptosis and G0/G1 cycle arrest

**Fig. 2:**
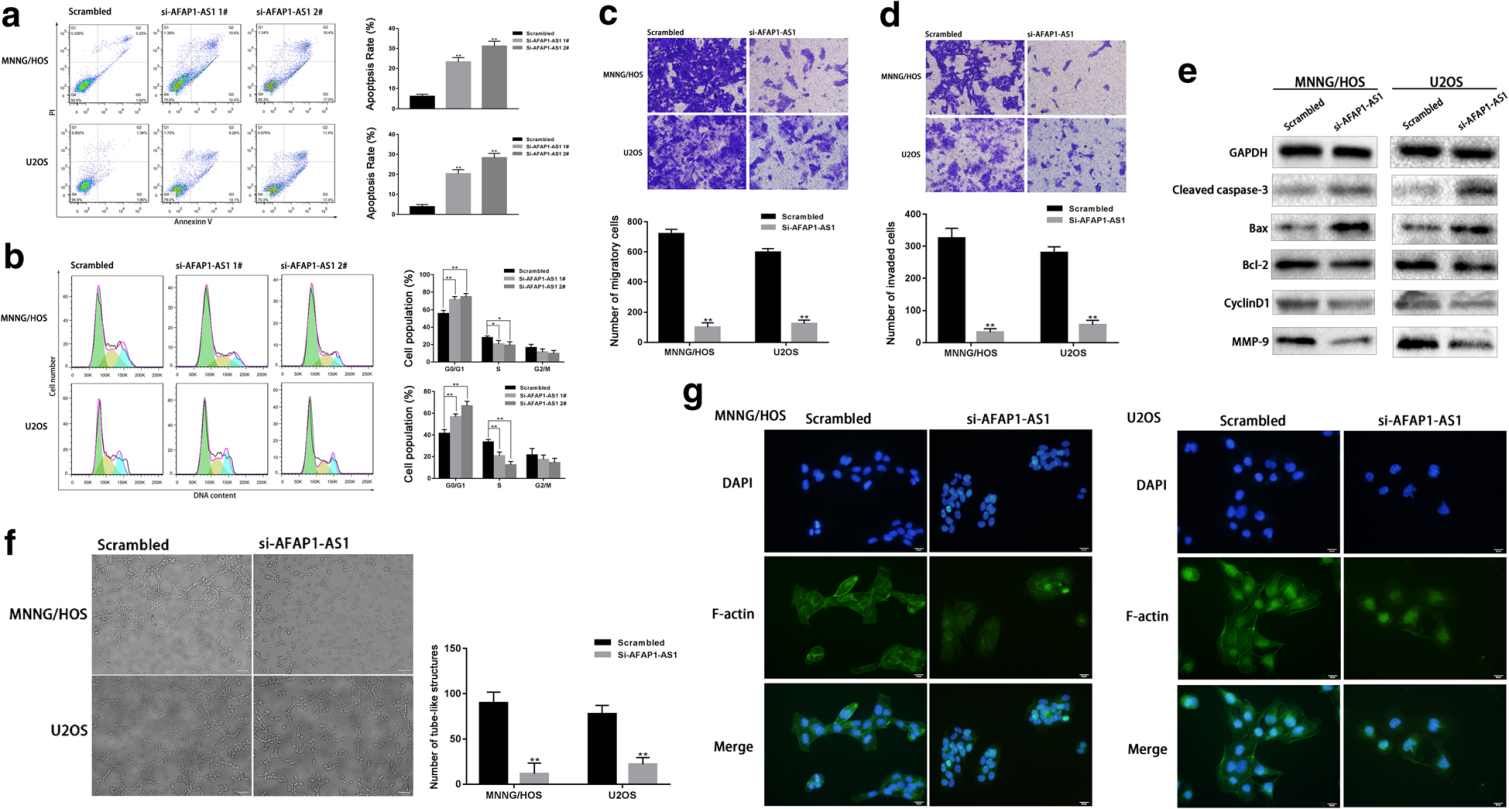
Effect of AFAP1-AS1 knockdown on the apoptosis, cell cycle, migration, invasion, actin filament integrity and vasculogenic mimicry formation of OS cells. **a** and **b** AFAP1-AS1 knockdown induced apoptosis and resulted in G0/G1 cell cycle arrest. **c** and **d** AFAP1-AS1 knockdown inhibited migration and invasion ability of OS cells. **e** In the AFAP1-AS1 knockdown group, the expression of cleaved Caspase 3, Bax were increased and the expression of Bcl-2, Cyclin D1 and MMP-9 were decreased compared to the scrambled group. **f** AFAP1-AS1 knockdown inhibited the VM formation ability of OS cells. **g** AFAP1-AS1 knockdown in OS cells induced loss of actin filament integrity. The integrity and fluorescence intensity of actin filament in osteosarcoma cells were obviously decreased. *P < 0.05, **P < 0.01

Flow cytometric analysis was applied to assess the effect of AFAP1-AS1 on cell apoptosis and cycle distribution in OS cells. Compared to control group, we observed that the apoptotic rate of OS cells was elevated significantly in AFAP1-AS1 knockdown group (Fig. 2a). Further, we examined the effect of AFAP1-AS1 knockdown on the cell cycle of OS cells by flow cytometry and found that AFAP1-AS1 knocked down cells presented an increased distribution in the G0/G1 phase and a decreased distribution in the S phase. No significant difference was observed in the G2/M phase. The cell cycle analysis suggested that knock down of AFAP1-AS1 in the OS cells resulted in G0/G1 cell cycle arrest and inhibition of S cell cycle progression (Fig. 2b). Altogether, these data suggested that AFAP1-AS1 knockdown-induced apoptosis and cell cycle arrest could result in the inhibition of OS cells proliferation in vitro. Besides, in the AFAP1-AS1 knockdown groups, we observed that the expression of cleaved Caspase 3, Bax were increased and the expression of Bcl-2 and Cyclin D1 were decreased (Fig. 2e).

### Knockdown of AFAP1-AS1 inhibited OS cells migration and invasion

To observe the effect of AFAP1-AS1 on cell invasive potential in OS cells, the migration and invasion assays using transwell was performed as described in Materials and Methods. We found that migrated and invaded OS cells in transwell assays were reduced obviously following AFAP1-AS1 knockdown (Fig. 2c and d), which suggested that knockdown of AFAP1-AS1 inhibited the migration and invasiveness of OS cells. In addition, we observed that the expression of MMP-9 was decreased in the AFAP1-AS1 knockdown groups (Fig. 2e).

### Knockdown of AFAP1-AS1 lowered the actin filament integrity in OS cell

In order to assess the impact of AFAP1-AS1 silencing on actin filament integrity of OS cells, We performed immunofluorescence assay in MNNG/HOS and U2OS cells using FITC-Phalloidin. No obvious morphological changes were observed under phase contrast microscopy between negative control and AFAP1-AS1 knocked down groups. However, compared to control group, the integrity and fluorescence intensity of actin filament in osteosarcoma cells were obviously decreased through immunofluorescence assay, demonstrating that AFAP1-AS1 knockdown in OS cells induced loss of actin filament integrity (Fig. 2g).

### Knockdown of AFAP1-AS1 decreased the vasculogenic mimicry formation in OS cell

Vasculogenic mimicry (VM) is the formation of micro vascular structures in malignant tumors and has been considered to be closely related with the growth, invasion, metastases of cancer cells. To investigate whether AFAP1-AS1 impact the formation of VM in OS cells, we performed the tube formation assay. Compared to the control group, apparent decreased number of tube-like structures were observed in AFAP1-AS1 knock downed OS cells, which demonstrating that AFAP1-AS1 knockdown could inhibit the VM formation capacity of OS cells and AFAP1-AS1 might play an important role in VM formation of OS cells (Fig. 2f).

### Regulating role of AFAP1-AS1 on AFAP1

Since AFAP1-AS1 is transcribed from the antisense strand of AFAP1 gene, qRT-PCR and Western blot were conducted to examine the expression change of AFAP1 following knockdown of AFAP1-AS1. However, no significant change of AFAP1 expression was observed when AFAP1-AS1 was knocked down in MNNG/HOS and U2OS cells. This result suggested that the cell genotype and behaviors of OS cells were impacted by AFAP1-AS1 without interfering of AFAP1 expression (Additional file 1: Figure S1).

### Knockdown of AFAP1-AS1 inhibited tumor growth in vivo

**Fig. 3:**
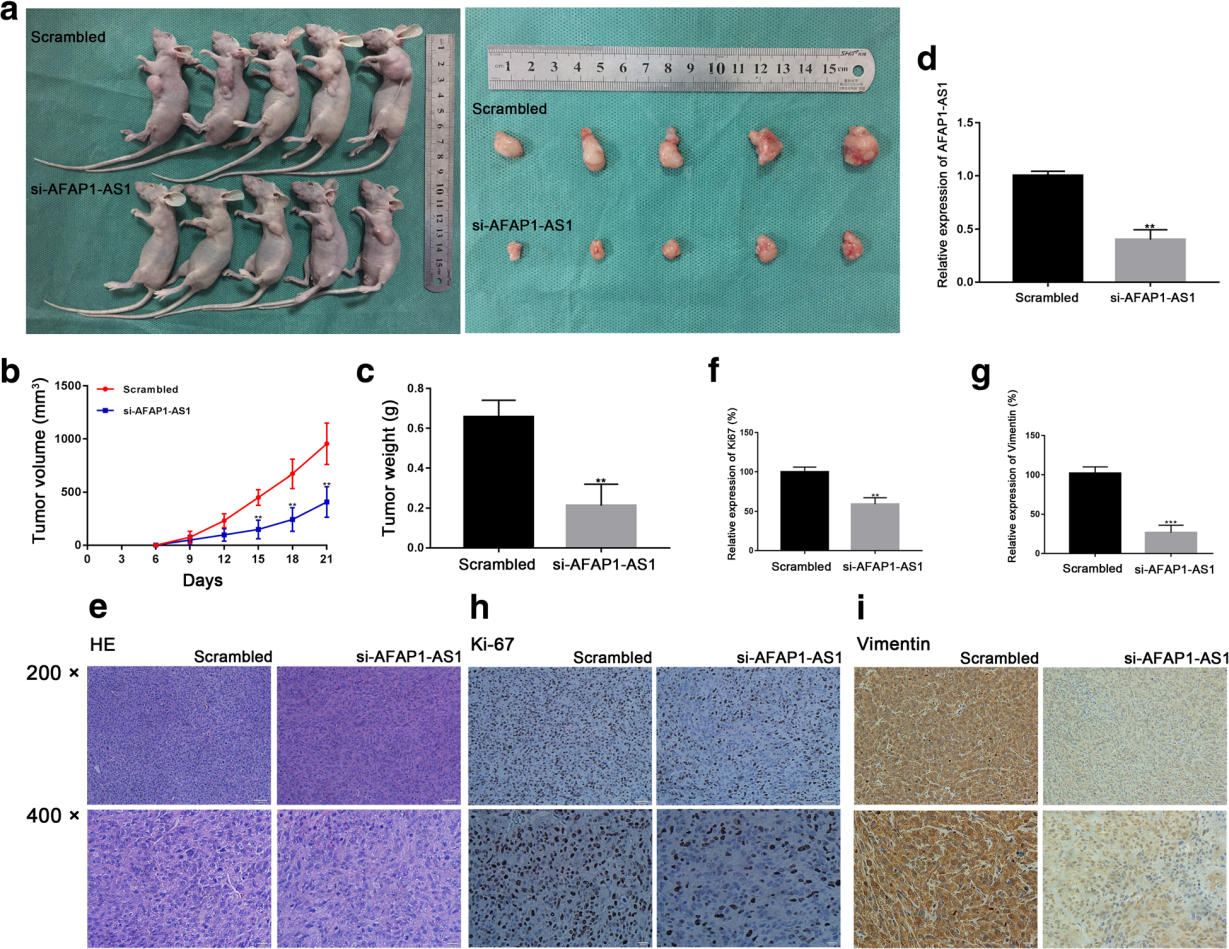
Effect of AFAP1-AS1 knockdown on osteosarcoma in vivo. **a**, **b** and **c** The volume and weight of tumor xenograft in nude mice with AFAP1-AS1 knockdown was reduced compared to the scrambled nude mice. Tumor xenograft growth in the AFAP1-AS1 knockdown nude mice was slower than that in the control group. **d** The average expression of AFAP1-AS1 was decreased in nude mice with AFAP1-AS1 knockdown compared to the scrambled nude mice. **e**, **h** and **i** The tumor tissues were under HE and IHC staining using antibodies against Ki-67 and vimentin. **f** and **g** IHC analysis demonstrated a significant decrease in average Ki67 and vimentin expression in the AFAP1-AS1 knockdown group compared to that of the scrambled group. *P < 0.05, **P < 0.01, ***P < 0.001

To further examine the oncogenic role of AFAP1-AS1 in the OS tumorigenesis, a xenograft mouse models was constructed through injecting MNNG/HOS cells subcutaneously into BALB/c nude mice as described in the materials and methods section. After 16 days, the tumors were harvested. During the whole tumor growth period, both the tumor weight and volume increased slowly in si-AFAP1-AS1 group compared to the control group (Fig. 3a, b and c). The average expression of AFAP1-AS1 in xenograft tumors was lower in the si-AFAP1-AS1 group than the control group (Fig. 3d). Moreover, the result of IHC demonstrated that the tumors collected from the si-AFAP1-AS1 group presented obviously lower Ki67 and vimentin expression compared to tumors in the control group (Fig. 3f, g, h and i). Taken together, our data suggested that AFAP1-AS1 knockdown could suppress tumor growth of osteosarcoma in vivo. The decrease of vimentin expression also indicated that AFAP1-AS1 knockdown suppressed osteosarcoma invasiveness capability.

### Knockdown of AFAP1-AS1 inhibited EMT and Twist1 expression in OS cells

**Fig. 4:**
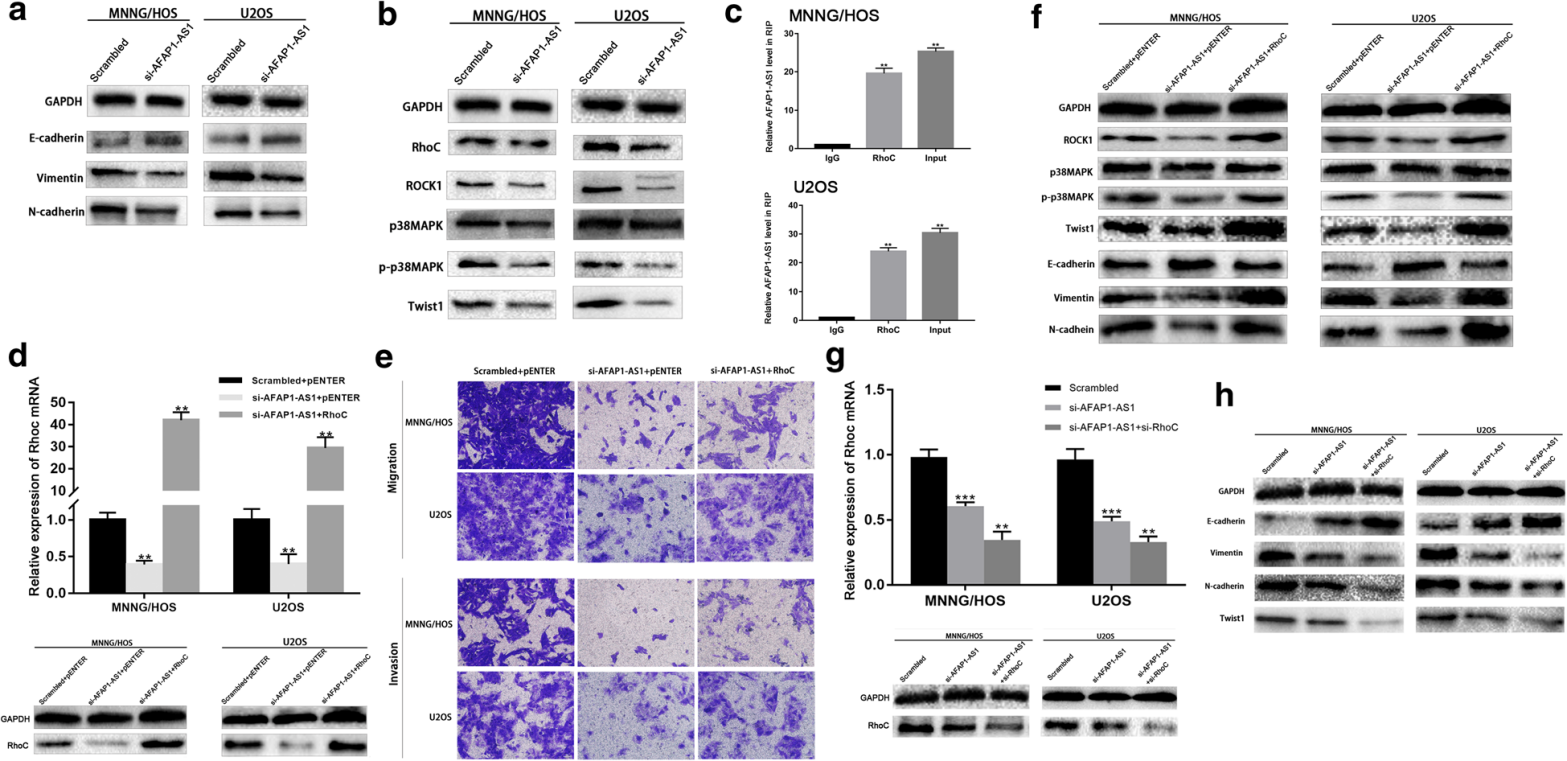
Effect of AFAP1-AS1 knockdown on molecular expression of OS cells and AFAP1-AS1 knockdown-inhibited EMT is mediated via RhoC/ROCK1/p38MAPK//Twsit1 signaling pathway. **a** AFAP1-AS1 knockdown in OS cells led to significantly decreased expression of mesenchymal markers (N-cadherin and Vimentin) and increased expression of epithelial marker E-cadherin. **b** In the AFAP1-AS1 knockdown group, the expression of RhoC, ROCK1, p-p38MAPK and Twsit1 was decreased compared to the scrambled group. **c** RIP assay demonstrated that AFAP1-AS1 interacted with RhoC in OS cells. **d**, **e** and **f** Overexpression of RhoC in AFAP1-AS1 knockdown OS cells could rescue AFAP1-AS1 downregulation-induced inhibition of cell migration, invasion, EMT, and the expression level of ROCK1, phosphorylated p38MAPK and Twist1 were rescued. **g** and **h** Both downregulating AFAP1-AS1 and RhoC, the expression of Twist and EMT of OS cells were inhibited further. *P < 0.05, **P < 0.01, ***P < 0.001

In various malignant tumors including OS, EMT plays a critical role in local invasion and distant metastasis. Knockdown of AFAP1-AS1 in both MNNG/HOS and U2OS cells led to significantly decreased expression of mesenchymal markers (N-cadherin and Vimentin) and increased expression of epithelial marker E-cadherin (Fig. 4a). To further explore the mediating mechanism, we aimed at the regulatory effect of AFAP1-AS1 on EMT-related transcription factors. Accumulating evidence showed that transcription factors of the Twist family played pivotal roles in EMT progression. Here we found that knockdown of AFAP1-AS1 resulted in significantly decreased expression of Twist1 in OS cells (Fig. 4b). Taken together, knockdown of AFAP1-AS inhibited EMT in OS cells and reduced expression of EMT-related transcription factor Twist1.

### Knockdown of AFAP1-AS1 inhibited EMT via RhoC/ROCK1/p38MAPK signaling pathway

Rho/ROCK signaling have been reported to play important roles in EMT regulation in several malignancies in previous studies. We examined Rho/ROCK signaling in OS cell lines after downregulation of AFAP1-AS1. Our results indicated that knockdown of AFAP1-AS1 led to decreased expression levels of RhoC and ROCK1 in osteosarcoma cell lines. In addition, we observed a significantly decrease of the expression level of phosphorylated p38MAPK, which has been reported to be a downstream signaling of Rho/ROCK signaling and regulate expression of Twist1 in various cancers (Fig. 4b). Our results indicated that knockdown of AFAP1-AS1 inhibited RhoC/ROCK1/p38MAPK signaling pathway.

### AFAP1-AS1 interacted with RhoC in OS cells

To explore the mechanism by which AFAP1-AS1 regulates RhoC/ROCK1/p38MAPK/Twist1 signaling pathway in OS cells, we examined whether AFAP1-AS1 interact with RhoC in OS cells. We conducted RNA immunoprecipitation (RIP) assay which demonstrated that AFAP1-AS1 bind to RhoC in both MNNG/HOS and U2OS cell lines (Fig. 4c). In addition, overexpression of RhoC via transfecting RhoC cDNA expression plasmid could rescue AFAP1-AS1 downregulation-induced inhibition of cell migration, invasion and EMT. Besides, the expression level of ROCK1, phosphorylated p38MAPK and Twist1 were also elevated (Fig. 4d, e and f). In together, our results indicated that the AFAP1-AS1 downregulation-inhibited EMT is mediated by the RhoC/ROCK1/p38MAPK/Twist1 signaling pathway. Moreover, we carried out additional experiment by both downregulating AFAP1-AS1 and RhoC, the expression of Twist and EMT of OS cells were inhibited further (Fig. 4g and h). Combined with the results above, RhoC could be considered the key underlying target of AFAP1-AS1.

### Knockdown of AFAP1-AS1-inhibited EMT is mediated by the Twsit1

**Fig. 5:**
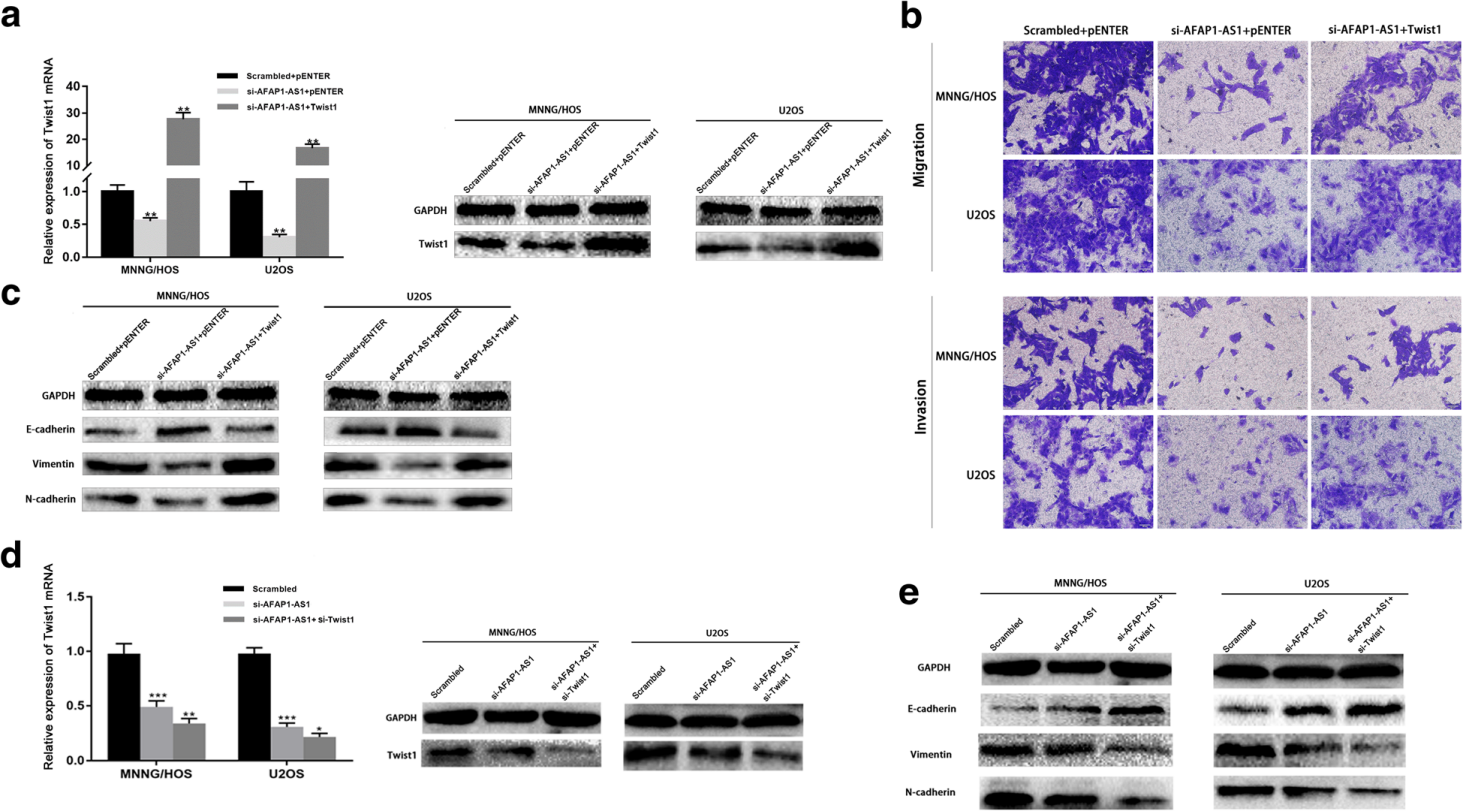
Knockdown of AFAP1-AS1-inhibited EMT is mediated by the Twsit1. **a**, **b** and **c** Overexpression of Twist1 in AFAP1-AS1 knockdown OS cells could rescue AFAP1-AS1 downregulation-induced inhibition of cell migration, invasion and EMT. **d** and **e** Both downregulating AFAP1-AS1 and Twist1, EMT of OS cells were inhibited further. *P < 0.05, **P < 0.01, ***P < 0.001

To confirm the involvement of Twist1 in AFAP1-AS1 downregulation-suppressed EMT, Twist1 was over-expressed in AFAP1-AS1 downregulated OS cells through co-transfection using Twist1 cDNA expression plasmid and si-AFAP1-AS1. Our results revealed that transfection with Twist1 cDNA expression plasmid significantly promoted si-AFAP1-AS1-induced inhibition of cell migration, invasion and EMT, as indicated by an increased expression of mesenchymal cell markers (N-cadherin, Vimentin) and a decreased expression of epithelial cell markers (E-cadherin) in osteosarcoma cell lines (Fig. 5a, b and c). Besides, by both downregulating AFAP1-AS1 and Twist1, EMT of OS cells were inhibited further (Fig. 5d and e). These results indicated that AFAP1-AS1 downregulation-inhibited EMT is mediated by Twist1.

## Discussion

Recently, an increasing number of studies have presented the pivotal roles of lncRNAs during the occurrence and development of malignancies. With regard to osteosarcoma, lncRNAs such as HOTAIR, HULC, H19 and MALAT-1, have been identified to be aberrantly expressed in cancer and function with underlying mechanisms [18, 25]. In the present study, we demonstrated the evidence that AFAP1-AS1 was significantly overexpressed in OS tissue and cell lines. In consistent with previous studies of AFAP1-AS1 in other types of cancer, both the results in vivo and vitro in our present study indicated that AFAP1-AS1 exerts oncogenic roles in development and progression of osteosarcoma. Thus AFAP1-AS1 could be considered as a potential therapeutic targets of OS.

It should be noted that Li et al.’s study has reported AFAP1-AS1 was overexpressed in osteosarcoma and high expression of AFAP1-AS1 was associated with poor prognosis of osteosarcoma patients [24]. In their study, AFAP1-AS1 was demonstrated to play an oncogenic role through regulating miR-4695-5p/TCF4-β-catenin signaling. However, further functional roles and underlying regulatory mechanism of AFAP1-AS1 have not been investigated in osteosarcoma. Compared to Li et al.’s study, we aimed on the interacting target of AFAP1-AS1 and performed in-depth investigation relatively.

LncRNAs were reported to play pivotal roles in tumorigenesis by a variety of modes such as chromatin modification, transcriptional interference, protein activity or localization modulating, miRNAs sponging. AFAP1-AS1, as an antisense transcript lncRNA, is transcribed from the AFAP1 gene in its antisense direction, containing several overlapping and complementary regions. According to existing viewpoints, antisense transcript lncRNAs appear to exert in-cis (affecting genes on the same chromosome where they are transcribed) or in-trans (affecting genes on another chromosome) effects in a highly cell-specific manner [26–28]. Previous researches have investigated the association between AFAP1-AS1 and AFAP1 in several types of tumor, among which contradictory findings were presented in these studies [29–31]. In our study, no significant expression change was found in either AFAP1 mRNA or protein when AFAP1-AS1 was downregulated, which preliminarily suggested that AFAP1-AS1 exerted no regulatory effect on AFAP1 gene in osteosarcoma cells.

In addition to the investigations in aspects of cell apoptosis, cell cycle arrest, migration and invasiveness capability, our study focused on the effect of AFAP1-AS1 on EMT. Accumulated evidence indicated that EMT promotes aggressive tumor phenotypes that leads to local invasion and distant metastasis through lymph and blood circulation. Numerous studies revealed that tumor invasion and metastasis can presumably be suppressed by targeting EMT-regulating factors [6, 32]. Thus targeting EMT process is presently considered to be a promising strategy to inhibit metastasis and improve cancer patients’ survival. However, the detailed regulatory mechanism of EMT remains unclear, especially in specific tumor type.

In the present study, we determined that AFAP1-AS1 downregulation resulted in EMT inhibition in osteosarcoma and furtherly explore related mechanism. Twist1 was reported to be a molecular regulator that down-modulate epithelial genes and up-modulate mesenchymal genes. Activated Twist upregulates N-cadherin and downregulates E-cadherin, which are the hallmarks of EMT. Besides Twist also protects cancer cells from apoptotic cell death. As a EMT-related transcriptional factor, Twist1 has been found to be overexpressed in numerous malignancies. Studies have presented that targeted downregulation of Twist1 can reverse EMT [33–36]. In our study, AFAP1-AS1 knockdown caused upregulation of E-cadherin expression and downregulation of N-cadherin and Vimentin expression, which providing an evidence of EMT inhibition. Moreover, AFAP1-AS1 knockdown inhibited EMT by reducing Twsit1 expression.

We further explore the intermediate regulation mechanism between AFAP1-AS1 and EMT, mainly in aspect of Rho GTPases and related signaling pathway. Rho GTPases and their downstream signaling were demonstrated in numerous studies to play critical roles in regulation of tumor angiogenesis, invasion, metastasis and EMT via complicated mechanisms. Studies have also explored the role of ROCK1, a pivotal downstream target of Rho GTPases. Increased expression of Rho GTPases and ROCK1 were often observed in cancers [37–39]. Besides according to Zhang et al.’s study [27], AFAP1-AS1 was proved to play oncogenic role in hepatocellular carcinoma via Rho GTPases related signaling. Through bioinformatics tool CATRAPID omics [40], we found that there was high interaction propensity between lncRNA AFAP1-AS1 and protein RhoC. In addition, another bioinformatics tool RPISeq [41] also showed strong positive interaction prediction between them. In addition, RhoC was reported to be highly expressed in human osteosarcoma and associated with patients’ poor prognosis [42]. P38MAPKs, are a class of mitogen-activated protein kinases that participate in various essential cell behaviors such as inflammation responding, apoptosis, autophagy, senescence and differentiation. EMT, was also demonstrated to be regulated by p38MAPKs and their relevant upstream or downstream signaling cascades. Studies reported that EMT-related transcriptional factors such as Twist1 could be regulated by p38MAPKs. Activated Rho/ROCK1 signaling could promote EMT, in which p38MAPK can act as the intermediate signaling target [43–48]. In our study, AFAP1-AS1 knockdown downregulated the expression levels of RhoC, ROCK1, phosphorylated p38MAPK, and Twist1 in osteosarcoma cells.

Specific lncRNAs can exert their function through interacting with various RNA binding proteins (RBPs) and leading to regulation of gene expression via chromosome reprogramming, DNA methylation, and protein modification. Studies also showed that lncRNAs can combine with specific proteins, modulate their activity, and even alter cellular localization of these protein [15, 18]. In our present study, based on existing evidence, we further affirmed that AFAP1-AS1 binds to RhoC in OS cells and exert regulating function by influencing RhoC-mediated signaling pathways.

Finally, the limitation of present study should be pointed out. We included osteosarcoma patients’ tumor specimens and clinical information in our study. However, due to limited follow-up time, information about response to adjuvant chemotherapy, recurrence, metastasis rates and overall survival were unable to be collected. These data would be valuable to assess the importance of AFAP1-AS1 in osteosarcoma. Besides, the potential regulatory mechanism of AFAP1-AS1 in OS could be investigated in more depth.

## Conclusion

This study demonstrates lncRNA AFAP1-AS1 is overexpressed in osteosarcoma and plays an oncogenic role in osteosarcoma through RhoC/ROCK1/p38MAPK/Twist1 signaling pathway, in which RhoC acts as the interaction target of AFAP1-AS1. Our findings indicated a novel molecular mechanism underlying the tumorigenesis and progression of osteosarcoma. AFAP1-AS1 could serve as a promising therapeutic target in OS treatment.

## Additional files

Additional file 1: **Figure S1.** Knockdown of AFAP1-AS1 exerted no significant alteration on AFAP1 mRNA and protein expression. (TIF 106 kb)

Additional file 2: The primer sequences for PCR and the sequences of siRNAs used in this study. (DOCX 15 kb)

Additional file 3: Clinicopathological characteristics of the patients enrolled in this study. (DOCX 16 kb)

## Data Availability

The datasets used and/or analyzed during the current study are available from the corresponding author on reasonable request.

## Abbreviations

AFAP1: Actin filament-associated protein 1
AFAP1-AS1: Actin filament-associated protein 1-antisense RNA 1
EMT: Epithelial-mesenchymal transition
IHC: Immunohistochemistry
lncRNA: Long non-coding RNA
MMP: Matrix metalloprotease
OS: Osteosarcoma
p- p38MAPKs: Phosphorylated p38 mitogen-activated protein kinases
p38MAPKs: p38 mitogen-activated protein kinases
qRT-PCR: Quantitative real time polymerase chain reaction
Rho: Ras homolog gene family member
RIP: RNA immunoprecipitation
ROCK: Rho-associated kinase
siRNA: Small Interfering Ribonucleic Acid
VM: Vasculogenic mimicry
