# Correction to: LncRNA AFAP1-AS1 promotes tumorigenesis and epithelial-mesenchymal transition of osteosarcoma through RhoC/ROCK1/p38MAPK/Twist1 signaling pathway

**DOI:** 10.1186/s13046-020-01574-2

**Published:** 2020-04-28

**Authors:** Deyao Shi, Fashuai Wu, Shidai Mu, Binwu Hu, Binlong Zhong, Feng Gao, Xiangcheng Qing, Jianxiang Liu, Zhicai Zhang, Zengwu Shao

**Affiliations:** 1 Department of Orthopaedics, Union Hospital, Tongji Medical College, Huazhong University of Science and Technology, 1277 Jiefang Road, Wuhan, 430022 China; 2 Institute of Hematology, Union Hospital, Tongji Medical College, Huazhong University of Science and Technology, 1277 Jiefang Road, Wuhan, 430022 China

**Correction to: J Exp Clin Cancer Res**

**https://doi.org/10.1186/s13046-019-1363-0**

In the original publication of this manuscript [1], Fig. 5a needs to be revised, and adjustments have also been made to the captions for Figs. 2, 4, 5 and S1 to improve clarity for the reader. The revised Fig. 5 and captions for Figs. 2,4 and S1 are shown below:

**Fig. 5:**
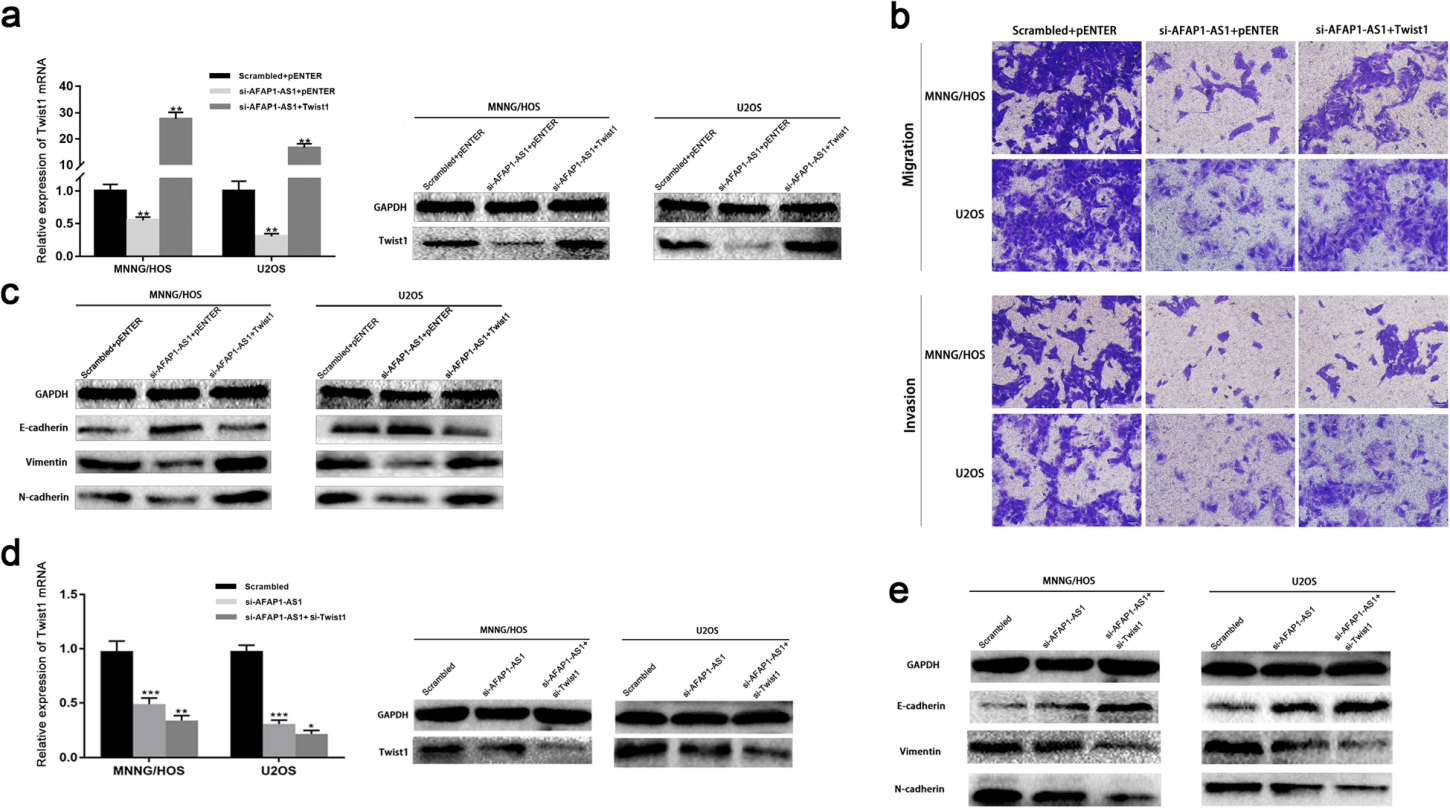
Knockdown of AFAP1-AS1-inhibited EMT is mediated by the Twsit1. **a**, **b** and **c** Overexpression of Twist1 in AFAP1-AS1 knockdown OS cells could rescue AFAP1-AS1 downregulation-induced inhibition of cell migration, invasion and EMT. **d** and **e** Both downregulating AFAP1-AS1 and Twist1, EMT of OS cells were inhibited further. *P < 0.05, **P < 0.01, ***P < 0.001. In Fig. 5a / Fig. 5c and Fig. 5d / Fig. 5e, the same GAPDH images were used to normalize in multiple panels under the same experimental treatments

Fig. 2 Effect of AFAP1-AS1 knockdown on the apoptosis, cell cycle, migration, invasion, actin filament integrity and vasculogenic mimicry formation of OS cells. a and b AFAP1-AS1 knockdown induced apoptosis and resulted in G0/G1 cell cycle arrest. c and d AFAP1-AS1 knockdown inhibited migration and invasion ability of OS cells. e In the AFAP1-AS1 knockdown group, the expression of cleaved Caspase 3, Bax were increased and the expression of Bcl-2, Cyclin D1 and MMP-9 were decreased compared to the scrambled group. f AFAP1-AS1 knockdown inhibited the VM formation ability of OS cells. g AFAP1-AS1 knockdown in OS cells induced loss of actin filament integrity. The integrity and fluorescence intensity of actin filament in osteosarcoma cells were obviously decreased. *P < 0.05, **P < 0.01. In Fig. 2e / Fig. 4a / Fig. 4b / Fig. S1, the same GAPDH images were used to normalize in multiple panels under the same experimental treatments.

Fig. 4 Effect of AFAP1-AS1 knockdown on molecular expression of OS cells and AFAP1-AS1 knockdown-inhibited EMT is mediated via RhoC/ROCK1/p38MAPK/Twsit1 signaling pathway. a AFAP1-AS1 knockdown in OS cells led to significantly decreased expression of mesenchymal markers (Ncadherin and Vimentin) and increased expression of epithelial marker E-cadherin. b In the AFAP1-AS1 knockdown group, the expression of RhoC, ROCK1, p-p38MAPK and Twsit1 was decreased compared to the scrambled group. c RIP assay demonstrated that AFAP1-AS1 interacted with RhoC in OS cells. d, e and f Overexpression of RhoC in AFAP1-AS1 knockdown OS cells could rescue AFAP1-AS1 downregulation-induced inhibition of cell migration, invasion, EMT, and the expression level of ROCK1, phosphorylated p38MAPK and Twist1 were rescued. g and h Both downregulating AFAP1-AS1 and RhoC, the expression of Twist and EMT of OS cells were inhibited further. *P < 0.05, **P < 0.01, ***P < 0.001. In Fig. 2e / Fig. 4a / Fig. 4b/ Fig. S1, Fig. 4f / Fig. 4d and Fig. 4g / Fig. 4h, the same GAPDH images were used to normalize in multiple panels under the same experimental treatments.

Figure S1. Knockdown of AFAP1-AS1 exerted no significant alteration on AFAP1 mRNA and protein expression. In Fig. 2e / Fig. 4a / Fig. 4b / Fig. S1, the same GAPDH images were used to normalize in multiple panels under the same experimental treatments.
