# Supplementary figures and images for: LncRNA AFAP1-AS1 promotes tumorigenesis and epithelial-mesenchymal transition of osteosarcoma through RhoC/ROCK1/p38MAPK/Twist1 signaling pathway
Source: J Exp Clin Cancer Res. 2019 Aug 23;38:375. doi: 10.1186/s13046-019-1363-0 (PMC6708246; doi:10.1186/s13046-019-1363-0)

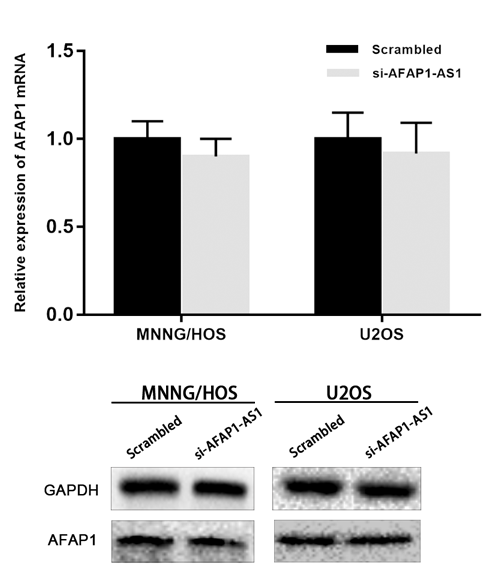

Supplement: Supplementary file 1 — Figure S1. Knockdown of AFAP1-AS1 exerted no significant alteration on AFAP1 mRNA and protein expression. (TIF 106 kb) [file 13046_2019_1363_MOESM1_ESM.tif]
