# Supplementary material for: LncRNA AFAP1-AS1 promotes tumorigenesis and epithelial-mesenchymal transition of osteosarcoma through RhoC/ROCK1/p38MAPK/Twist1 signaling pathway
Source: J Exp Clin Cancer Res. 2019 Aug 23;38:375. doi: 10.1186/s13046-019-1363-0 (PMC6708246; doi:10.1186/s13046-019-1363-0)
Supplement: Supplementary file 2 — The primer sequences for PCR and the sequences of siRNAs used in this study. (DOCX 15 kb) [file 13046_2019_1363_MOESM2_ESM.docx]

The primer sequences for PCR are as follows:

|  | forward | reverse |
| --- | --- | --- |
| AFAP1-AS1 | 5’-GAAGAGGCAACCAGGAGCG-3’ | 5’-GAAACTGAGGCACAGAGGGATT-3’ |
| AFAP1 | 5’-AGAGTGTCCTCCTCCACCAA-3’ | 5’-CTTGGCCTCTGATTTGGAAC-3’ |
| Twist1 | 5’-CGGACAAGCTGAGCAAGAT-3’ | 5’-CTGGAGGACCTGGTAGAGGA-3’ |
| RhoC | 5’-CTGAGAAGTGGACCCCAGAG-3’ | 5’-CAAGGTAGCCAA AGGCACTG-3’ |
| GAPDH | 5’-AATCCCATCACCATCTTCCAG-3’ | 5’-GAGCCCCAGCCTTCTCCAT-3’ |

The sequences of siRNAs that targeted AFAP1-AS1 and scrambled siRNA are as follows:

| si-AFAP1-AS1 1# | 5’-GGACCACUUUGGUGUAUCUTT-3’ |
| --- | --- |
| si-AFAP1-AS1 2# | 5’-GGGCUUCAAUUUACAAGCATT-3’ |
| si-AFAP1-AS1 3# | 5’-GGUGGAGAAUGAACAUUCUTT-3’ |
| si-AFAP1-AS1 4# | 5’-GUCCCAGCUUACACUUGUATT-3’ |
| si-RhoC | 5’-GUGCCUUUGGCUACCUUGATT-3’ |
| si-Twist1 | 5’-GGUGUCUAAAUGCAUUCAUTT-3’ |
| si-NC  (scrambled negative control siRNA) | 5’-UUCUCCGAACGUGUCACGUTT-3’ |
