# Supplementary material for: LncRNA AFAP1-AS1 promotes tumorigenesis and epithelial-mesenchymal transition of osteosarcoma through RhoC/ROCK1/p38MAPK/Twist1 signaling pathway
Source: J Exp Clin Cancer Res. 2019 Aug 23;38:375. doi: 10.1186/s13046-019-1363-0 (PMC6708246; doi:10.1186/s13046-019-1363-0)
Supplement: Supplementary file 3 — Clinicopathological characteristics of the patients enrolled in this study. (DOCX 16 kb) [file 13046_2019_1363_MOESM3_ESM.docx]

**Table S2 Eight Patients’ clinicopathological characteristics**

|  | Number | Percentage (%) |
| --- | --- | --- |
| **Age** |  |  |
| ≥25y | 3 | 37.5 |
| <25y | 5 | 62.5 |
| **Gender** |  |  |
| Male | 5 | 62.5 |
| Female | 3 | 37.5 |
| **Pathological type** |  |  |
| conventional osteosarcoma | 6 | 75 |
| talangiectatic osteosarcoma | 1 | 12.5 |
| parosteal osteosarcoma | 1 | 12.5 |
| **Primary origin** |  |  |
| Femur | 5 | 62.5 |
| Tibia | 2 | 25 |
| Fibula | 1 | 12.5 |
| **TNM staging** |  |  |
| Ⅰ | 2 | 25 |
| Ⅱ | 3 | 37.5 |
| Ⅲ | 1 | 12.5 |
| Ⅳ | 2 | 25 |
| **Grade** |  |  |
| Low-grade | 2 | 25 |
| High-grade | 6 | 75 |
| **Maximum diameter of tumor** |  |  |
| ≥8cm | 5 | 62.5 |
| <8cm | 3 | 37.5 |
| **Pre-operative chemotherapy** |  |  |
| Yes | 0 | 0 |
| No | 8 | 100 |
| **Post-operative chemotherapy** |  |  |
| Yes | 6 | 75 |
| No | 2 | 25 |
| **Operation method** |  |  |
| Limb-salvage | 5 | 62.5 |
| Amputation | 3 | 37.5 |
| **Metastasis at diagnosis** |  |  |
| Yes | 2 | 25 |
| No | 6 | 75 |

It should be noted that we only collected specimens from patients who have not received chemotherapy before operation. The conditions for these patients before operation includes the following points: 1) The tumor loads were too large at the time of initial treatment. 2) Operation method was determined to be amputation. 3) Due to poor response, pre-operative chemotherapy was terminated. 4) Due to intolerable side effects, pre-operative chemotherapy was terminated. 5) Pre-operative chemotherapy was refused because of patients’ personal financial situation.
